# Charge-reversal nanomedicine based on black phosphorus for the development of A Novel photothermal therapy of oral cancer

**DOI:** 10.1080/10717544.2021.1909176

**Published:** 2021-04-05

**Authors:** Zimu Li, Qingyun Liu, Yi Zhang, Yao Yang, Xiaomin Zhou, Wei Peng, Zhigang Liang, Xiaowei Zeng, Qiuxu Wang, Nansha Gao

**Affiliations:** a Institute of Pharmaceutics, School of Pharmaceutical Sciences (Shenzhen), Sun Yat-sen University, Shenzhen, China; b Stomatology Department of Shenzhen Second People’s Hospital, Shenzhen, China; c Department of Stomatology, Affiliated Zhongshan Hospital of Dalian University, Dalian, China

**Keywords:** Oral cancer, black phosphorus, photothermal therapy, pH-response, charge reversal

## Abstract

Driven by the lifestyle habits of modern people, such as excessive smoking, drinking, and chewing betel nut and other cancer-causing foods, the incidence of oral cancer has increased sharply and has a trend of becoming younger. Given the current mainstream treatment means of surgical resection will cause serious damage to many oral organs, so that patients lose the ability to chew, speak, and so on, it is urgent to develop new oral cancer treatment methods. Based on the strong killing effect of photothermal therapy on exposed superficial tumors, we developed a pH-responsive charge reversal nanomedicine system for oral cancer which is a kind of classic superficial tumor. With excellent photothermal properties of polydopamine (PDA) modified black phosphorus nanosheets (BP NSs) as basal material, then used polyacrylamide hydrochloride-dimethylmaleic acid (PAH-DMMA) charge reversal system for further surface modification, which can be negatively charged at blood circulation, and become a positive surface charge in the tumor site weakly acidic conditions due to the breaking of dimethylmaleic amide. Therefore, the uptake of oral cancer cells was enhanced and the therapeutic effect was improved. It can be proved that this nanomedicine has excellent photothermal properties and tumor enrichment ability, as well as a good killing effect on oral cancer cells through in vitro cytotoxicity test and *in vivo* photothermal test, which may become a very promising new model of oral cancer treatment.

## 1. Introduction

Oral cancer is one of the most common malignant tumors in the head and neck, and the number of patients with oral cancer has increased dramatically in recent years (Izumchenko et al., 2020). At present, clinical treatment is mainly based on surgery (Marcazzan et al., 2018). However, to prevent the spread of cancer cells, complete tumor resection is required. Therefore, a large number of patients' entire tongues or gums have been removed as well as faces have been cut, which may lead to the loss of language, masticatory, gustation, and other basic functions of the patient, and bring a very serious impact on the patient's life (Huang & O’Sullivan, 2013; Sun et al., 2020). So it is urgent to develop non-operative oral cancer treatment methods to ensure the treatment effect and the quality of life of patients to the maximum extent.

In the past few decades, with the rapid development of nanotechnology and the mutual integration with biomedicine, there has been a large number of nanomedicine-platform research for the treatment of various diseases (Sahle et al., 2019; Li et al., 2020; Zhou et al., 2020; Liu et al., 2021). Especially in the field of tumor treatment (Tao et al., 2018; Dang & Guan, 2020; Lan et al., 2020), the model including breast (Zeng et al., 2018), lung (Gao et al., 2020), cervical (Duo et al., 2018), prostate (Zeng et al., 2019), pancreatic cancer (Banstola et al., 2019) and so on have achieved very good therapeutic effect. At the same time, the construction of nanomedicine-platform can integrate and apply new tumor treatment methods, including photothermal therapy (Cheng et al., 2017; Liu et al., 2018), photodynamic therapy (Zeng et al., 2020), immunotherapy (Liang et al., 2020), and so on, which broadens the thinking of tumor treatment and has the opportunity to greatly improve the cure rate of clinical malignant tumors. In particular, photothermal therapy has been proved to have a strong killing effect on superficial local solid tumors, but it is still not well applied in the treatment of many deep organ tumors due to the limited penetration depth of near-infrared (NIR) light (Zhang et al., 2019). However, the vast majority of oral cancers are originated from epithelial and mucosal mutations (Scully & Bagan, 2009), which occur in the exposed parts of the oral cavity, and it is extremely beneficial for the application of photothermal therapy. Besides, oral cancer usually has a long period of oral ulcers, oral lumps, and other obvious precancerous lesions (Brandizzi et al., 2008), which can be detected early. Therefore, the strong killing of local tumor cells through photothermal therapy is likely to achieve a radical cure for oral cancer in the early stage.

Black phosphorus nanosheets (BP NSs), as a new two-dimensional material, has attracted great attention from scholars all over the world in recent years (Tao et al., 2019; Tang et al., 2020) due to their superior photothermal conversion efficiency (Hu et al., 2020; Kong et al., 2020; Ouyang et al., 2020) and extremely low toxicity (Tao et al., 2017; Gao et al., 2019; Luo et al., 2019). However, BP NSs lack stability in air or water solution and are easy to be degraded, thus reducing their photothermal properties (Zeng et al., 2018). At the same time, due to the negative charge on the surface of BP NSs, the uptake efficiency in tumor sites is low, which also hinders their further application.

Dimethylmaleic amide, due to its adjacent carboxyl group can attack the amide bond and produce an unstable five-member ring intermediate, which can complete the amide bond breaking in a weakly acidic environment (Du et al., 2018). Before the amide bond breaks, the carboxyl group with a negative charge is exposed to the outside, but after the break, it becomes the amino group with a positive charge. Therefore, the surface modification of nanomedicine with dimethylmaleic acid (DMMA) can produce the effect of charge reversal in a weakly acidic environment.

**Figure 1.**
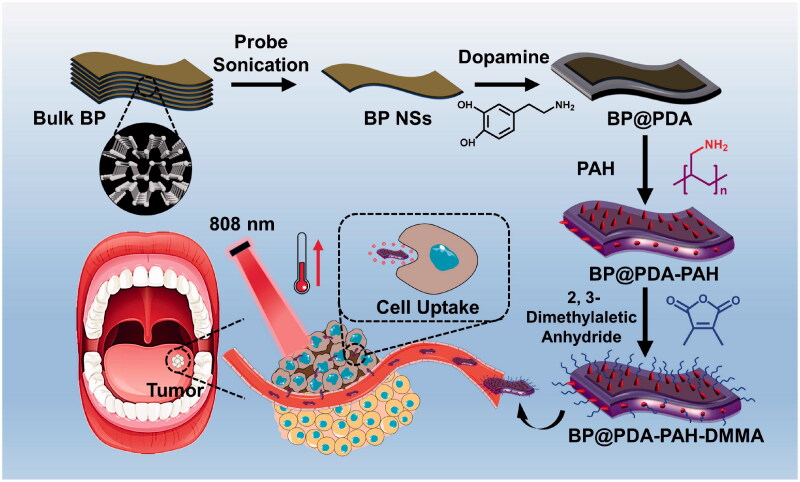
Schematic diagram of the preparation and treatment process of BP@PDA-PAH-DMMA nanomedicine for oral cancer therapy.

In this study, we constructed a charge reversal nanoplatform based on BP NSs for the treatment of oral cancer (Figure 1). BP NSs were first coated with polydopamine, which not only enhanced photothermal properties but also greatly improved their stability (Cheng et al., 2019; Wang et al., 2020). Moreover, surface modification of polyacrylamide hydrochloride (PAH) gives the nanoplatform a surface positive charge. After further connecting DMMA, the positive charge produced by amino groups of PAH was shielded, constituting the PAH-DMMA charge reversal system. This enables it to maintain surface negative charge during blood circulation, and form surface positive charge through dimethylmaleic amide fracture at the tumor site, to promote the enrichment and uptake of the nanomedicine at the tumor site, and kill oral cancer cells through good photothermal properties.

## 2. Materials and methods

### 2.1. Materials

Bulk BP was obtained from Guangxi Sino-Lin chem International, Inc (China), and persevered in the 4 °C refrigerator. Dopamine hydrochloride was purchased from Sigma-Aldrich (St. Louis, MO, USA). Polyallylamine hydrochloride (PAH) was provided by Ailin Chemical Technology Co., Ltd. Dimethyl sulfoxide (DMSO) was bought from Innochem Technology Co., Ltd (Beijing). 2, 3-dimethylaletic anhydride, 1-methyl-2-pyrrolidinone (NMP), triethylamine, and trihydroxy methyl aminomethane hydrochloride (Tris-HCl) were purchased from Aladdin Biochemical Technology Co., Ltd (Shanghai). HyPure Molecular Biology Grade Water was used to prepare all solutions. The other agents were used of the highest quality commercially available.

### 2.2. Preparation of BP NSs

The BP NSs were prepared using a simple liquid exfoliation of the corresponding bulk BP sample. Briefly, 50 mg of bulk BP was dispersed in 100 mL NMP, which is applied to reduce the oxidation of BP in exfoliation. The mixture solution was sonicated in an ice water bath for 8 h (amplifier: 35%, on/off cycle: 5 s/5 s). Ice water is used to keep the system at a relatively low temperature. Subsequently, the dispersion was centrifuged at 7000 rpm for 10 min to separate the unexfoliated bulk BP. Then, the supernatant was centrifuged at 15,000 rpm at 10 min to remove the BP quantum dots. The sediment was collected and kept under 4 °C for further experiments.

### 2.3. PDA coating on BP NS’s surface

2 mg BP NSs were dispersed in 2 mL Tris-HCl buffer. The pH of the solution was adjusted to 8.5 by sodium hydroxide and 1 mg of dopamine hydrochloride was added into the solution. Afterward, the solution was stirred for 6 h in the dark environment. The BP@PDA was gained by centrifuging at 15,000 rpm for 10 min and washed with water several times.

### 2.4. Conjugation of PAH onto the surface of BP@PDA

The above product BP@PDA was suspended in 2 mL Tris-HCl buffer (pH 8.5, 10 mM). 2 mg PAH was added into the solution and stirred for 12 h in the dark environment. Then the BP@PDA-PAH NSs were centrifuged at 15,000 rpm for 10 min and washed with water several times.

### 2.5. Preparation of BP@PDA-PAH-DMMA

Briefly, the above product BP@PDA-PAH was dispersed in 2 mL DMSO, as well as 60 μL triethylamine and 3 mg of 2, 3-dimethylmaleic anhydride were added. The mixture was stirred drastically for 24 h in dark. Finally, the BP@PDA-PAH-DMMA NSs were obtained by centrifugation at 15,000 rpm for 10 min and washed with water several times.

### 2.6. Characterizations of BP NSs and modified BP NSs

Transmission electron microscopy (TEM) images were obtained by JEM-2100UHR transmission electron microscopy. Different samples of BP NSs were dropped onto copper grid-coated carbon membranes and naturally air-dried. X-ray photoelectron spectroscopy (XPS) was operated with an X-ray photoelectron spectrometer (Nexsa, Thermo Scientific, United Kingdom). The size and zeta potential of different BP NSs were measured by nanoparticle size and Zeta potential analyzer (NanoBrook 90plus PALS, United States). All experiments were repeated three times independently and averaged.

### 2.7. Photothermal performance of different BP NSs

The photothermal properties of different BP NSs were evaluated by recording the temperature changes under 808 nm NIR laser irradiation (BOHR-808-FCIR8, Xi’an Bohr Optoelectronics Technology Co., Ltd., China). To evaluate the photothermal effect after different substances modification onto BP NSs, BP NSs, BP@PDA, and BP@PDA-PAH-DMMA of 100 μg·mL^–1^ BP concentration was irradiated with a laser power density of 1.0 W·cm^–2^ for 10 min. After that, the BP@PDA-PAH-DMMA of 50, 100, 150, and 200 μg·mL^–1^ were irradiated with the laser power density of 1.0 W·cm^–2^ for 10 min. And then, 100 μg·mL^–1^ of BP@PDA-PAH-DMMA were irradiated with laser power density of 0.5 W·cm^–2^, 1.0 W·cm^–2^, 1.5 W·cm^–2^, 2.0 W·cm^–2^ for 10 min respectively. Finally, BP@PDA-PAH-DMMA (100 μg·mL^–1^) was irradiated with the laser power density of 1.0 W·cm^–2^ in five repeated cycles of 10 min irradiation ON and 10 min OFF. An infrared thermal imaging camera was applied to record the temperature changes of NSs (Ti450, Fluke, United States).

### 2.8. Cell culture assays

Oral cancer cell line CAL-27 and SAS were selected for the research. The two kinds of cells were incubated in Dulbecco’s modified Eagle’s medium (DMEM) mixed with 10% (v/v) fetal bovine serum (Gibco), antibiotics penicillin (100 U·mL^–1^), and streptomycin (100 μg·mL^–1^) in a 5% CO_2_-humidified atmosphere at 37 °C.

### 2.9. Cellular uptake of nanomedicines

CAL-27 and SAS cells were cultured (1 × 10^6^ cells per dish) into 20 mm glass-bottomed Petri dishes at 37 °C for 24 h. DOX labeled BP@PDA-PAH-DMMA and BP@PDA-PEG were added into wells at a pH of 6.5 or 7.4. After incubation at 37 °C for 4 h, the cells were washed three times with PBS. Next, cells were treated with 4% paraformaldehyde solution for 15 min and then stained with 4′,6-diamidino-2-phenylindole (DAPI) for 10 min before CLSM analysis. The cells were observed with a confocal laser scanning microscope (LSN880, Zeiss, Germany) at 488 and 590 nm, as the excitation and emission wavelengths of DOX, as well as 358 and 461 nm, as the excitation and emission wavelengths of DAPI.

### 2.10. Cytotoxicity assay

The cytotoxicity of BP, BP@PDA, and BP@PDA-PAH-DMMA was assessed by the CCK-8 assay. In brief, CAL-27 and SAS cells were seeded into a 96-well plate at a density of 1 × 10^4^ and cultured for 24 h. After cells attachment, the medium was replaced with 100 μL of fresh medium containing 6.25, 12.5, 25, 50, 100 μg·mL^–1^ of BP, BP@PDA, BP@PDA-PAH, and BP@PDA-PAH-DMMA. After further 4 h incubation, different concentrations of groups were irradiated with an 808 nm laser (1.0 W·cm^–2^) for 10 min. After further 20 h incubation, 10 μL of CCK-8 were added into wells. After 2 h incubation, cell viability was determined to measure the absorbance at 450 nm by a multi-mode board reader (Victor Nivo, Wuxi Webforce Technology Co., Ltd, China).

### 2.11. *In vitro* safety assessment of different BP NSs

To evaluate the safety of the different BP NSs, CAL-27 and SAS cells were plated into 96-well plate for 24 h (37 °C, 5% CO_2_). Then, the old medium was removed and washed three times with PBS. Afterward, 100 μL of new fresh medium containing a series of concentrations of BP, BP@PDA, BP@PDA-PAH, and BP@PDA-PAH-DMMA (1, 10, 50, 100 μg·mL^–1^) After co-culturing for 24 h, 10 μL of CCK-8 was added. Approximately 2 h later, cell viability was detected using a multi-mode board reader (Victor Nivo, Wuxi Webforce Technology Co., Ltd, China) at 450 nm.

### 2.12. Tumor model establishment

The female BALB/c-nu/nu mice (4–6 weeks old) were purchased from the Laboratory Animal Center of Sun Yat-sen University (Guangzhou, China). All animal experimental protocols were given permission by the Administrative Committee on Animal Research in Sun Yat-sen University. In vivo experiments were all complied with the guidelines of the institutional animal ethics committee. CAL-27 cells suspension in PBS (about 2 × 10^6^ cells) were subcutaneously injected into the right flank area of the mice.

### 2.13. *In vivo* infrared thermal imaging

The CAL-27 tumor-bearing mice were intravenously injected with saline, BP@PDA-PEG, and BP@PDA-PAH-DMMA, respectively. And the dose of BP@PDA-PEG and BP@PDA-PAH-DMMA was 0.54 mg·kg^–1^ in 100 μL PBS. After 24 h, the mice were anesthetized and the tumor sites were irradiated with 808 nm NIR laser at the power density of 1.5 W·cm^–2^ for 5 min. An infrared thermal image camera was utilized to record the temperature changes every 30 s during the irradiation.

### 2.14. Statistical analysis

Unless otherwise stated, all experiments were performed at least in triplicate, and the data were represented as mean ± standard deviation. Statistical analysis was carried out by using SPSS 22.0 software for a one-way analysis of variance and subsequent Bonferroni test, with probability (*P*) less than 0.05 was considered statistically significant.

## 3. Results and discussion

### 3.1. Synthesis of BP@PDA-PAH-DMMA

The synthesis process of BP@PDA-PAH-DMMA can be seen in Figure 1, which can be divided into four steps. Firstly, the BP NSs with appropriate size and uniformity were prepared by liquid exfoliation and centrifugation. Secondly, in a weakly alkaline environment of pH 8.5, dopamine could form PDA through the reaction of catechol structure with other quinones or catechol structure after oxidation to quinones, as well as non-covalent bonding such as π-π stacking and hydrogen bond interaction, and then adhered to the surface of BP NSs to form a coating layer (Supplementary Figure S1). After that, the PAH is connected to BP@PDA through the Michael addition reaction between some amino groups of PAH and PDA. Finally, the reaction of 2, 3-dimethylmaleic anhydride and the residual amino group of PAH resulted in the carboxylation of the surface of the nanomedicine, so that BP@PDA-PAH-DMMA was obtained.

### 3.2. Morphology and characterizations

**Figure 2.**
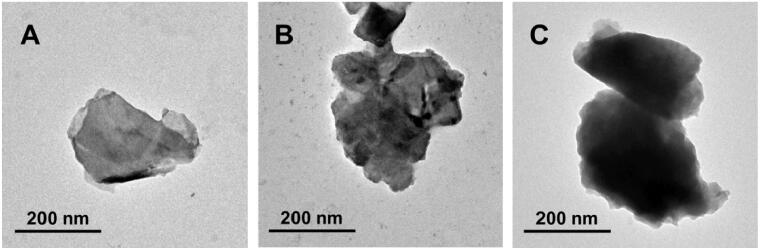
TEM images of (A) BP NSs; (B) BP@PDA; (C) BP@PDA-PAH-DMMA.

The morphology of BP NSs and the modified BP NSs were characterized by Transmission electron microscopy (TEM). As can be seen from TEM images (Figure 2(A–C)), the bare BP NSs obtained by the liquid exfoliation method are thin lamellar structures with a relatively smooth surface, but after the surface modification of PDA and PAH-DMMA, the surface of the nanosheets became rougher, and the thickness increased significantly. At the same time, it can be observed from TEM images that the size of different BP NSs is in the range of 200–350 nm, which is consistent with the results of dynamic light scattering (Supplementary Figure S2).

**Figure 3.**
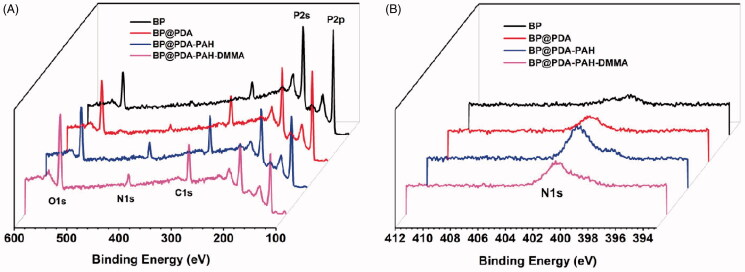
XPS spectra of BP NSs, BP@PDA, BP@PDA-PAH, and BP@PDA-PAH-DMMA: (A) Survey spectrum and (B) N1s spectrum.

The surface modification effect of BP NSs can be characterized by X-ray photoelectron spectroscopy (XPS). As can be seen from Figure 3, compared with BP NSs, the N1s (401.1 eV), C1s (284.6 eV), and O1s peaks (531.4 eV) of BP@PDA, BP@PDA-PAH, BP@PDA-PAH-DMMA was increased, which was consistent with the elements contained in PDA, indicating the successful coating of PDA on BP NSs surface. Compared with BP@PDA, the N1s and C1s peaks of BP@PDA-PAH also increased, which was in line with the elemental characteristics of PAH, proving that PAH was successfully modified on the surface of the PDA layer. Further, by comparing BP@PDA-PAH-DMMA and BP@PDA-PAH, it can be found that the increase of O1s peak accords with the elemental characteristics of DMMA, indicating that DMMA is successfully connected. Besides, the P2p peaks (129.2 eV) decreased gradually in the process of layer by layer modification, which was because the surface modification covered up the P element.

### 3.3. Effects of pH on zeta potential

**Figure 4.**
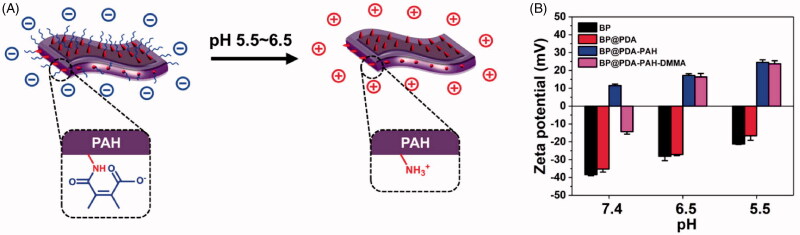
(A) Schematic diagram of pH-responsive charge reversal of BP@PDA-PAH-DMMA; (B) Surface zeta potential of BP NSs, BP@PDA, BP@PDA-PAH, and BP@PDA-PAH-DMMA at different pH of 7.4, 6.5, and 5.5.

Figure 4(A) is the schematic diagram of BP@PDA-PAH-DMMA to realize the tumor microenvironmental charge reversal. Due to the shedding of DMMA, the amino groups are exposed and the surface charge of the nanomedicine changes from negative to positive. The change of charge under different pH was analyzed by Zeta potential analyzer. It can be seen from Figure 4(B) and Table 1 that BP@PDA-PAH-DMMA has a negative charge on its surface under the condition of pH 7.4, while it has a positive charge on its surface under the weak acid environment of pH 6.5 and pH 5.5, indicating that this nanomedicine has a sensitive pH-responsive charge reversal ability. In contrast, BP NSs, BP@PDA, and BP@PDA-PAH maintain the same charge at all three pH conditions and cannot produce the effect of charge reversal.

**Table 1.** Surface zeta potentials of different NSs at different pH of 7.4, 6.5, and 5.5.

Samples (*n* = 3)	pH 7.4 (mV)	pH 6.5 (mV)	pH 5.5 (mV)
BP	–38.36 ± 0.65	–28.13 ± 2.43	–21.22 ± 0.28
BP@PDA	–35.26 ± 1.71	–27.11 ± 0.64	–16.57 ± 2.55
BP@PDA-PAH	11.55 ± 0.83	17.15 ± 1.01	24.54 ± 1.39
BP@PDA-PAH-DMMA	–14.32 ± 1.35	16.35 ± 1.96	23.70 ± 1.76

### 3.4. *In vitro* photothermal effects

**Figure 5.**
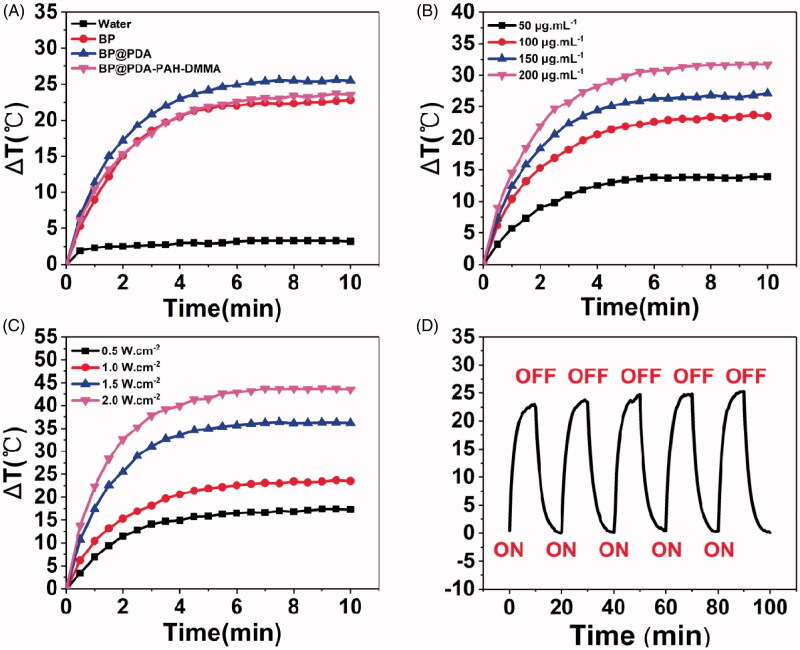
(A) Temperature rise curves of water, BP, BP@PDA, and BP@PDA-PAH-DMMA solution under 808 nm laser irradiation (1.0 W·cm^–2^) for 10 min. (B) Temperature rise curves of the BP@PDA-PAH-DMMA solution with different concentrations. (C) Temperature rise curves of the BP@PDA-PAH-DMMA solution under various irradiation power intensities. (D) The temperature change of BP@PDA-PAH-DMMA solution for five repeated laser on/off cycles with an 808 nm NIR laser at a power density of 1.0 W·cm^–2^.

The photothermal properties of the nanotherapeutic system were evaluated by temperature variations under 808 nm NIR laser irradiation for 10 min. Figure 5(A) shows the temperature rise of bare BP NSs, BP@PDA, BP@PDA-PAH, and BP@PDA-PAH-DMMA with both concentrations of 100 μg·mL^–1^ under the same irradiation conditions. It can be seen that due to the high photothermal conversion efficiency, bare BP NSs can produce a great temperature rise under irradiation (Δ*T* = 22.8 °C). Compared with the temperature change of bare BP NSs, the temperature rise value of BP@PDA (Δ*T* = 25.5 °C) was increased, which is also attributed to the good photothermal conversion efficiency of the PDA layer. Besides, as the material concentration and irradiation power change, the temperature rise will also change (Figure 5(B, C)). This reminds us that in clinical use, the concentration and power can be adjusted according to actual needs to produce a suitable temperature rise. Besides, there was no obvious difference in temperature rise after five irradiation cycles (Figure 5(D)), indicating that the nanotherapeutic platform had excellent irradiation stability. In general, the nanotherapeutic system was demonstrated to have excellent photothermal properties and irradiation stability *in vitro*, which meets the needs of clinical photothermal therapy.

### 3.5. Cellular uptake of NSs

**Figure 6.**
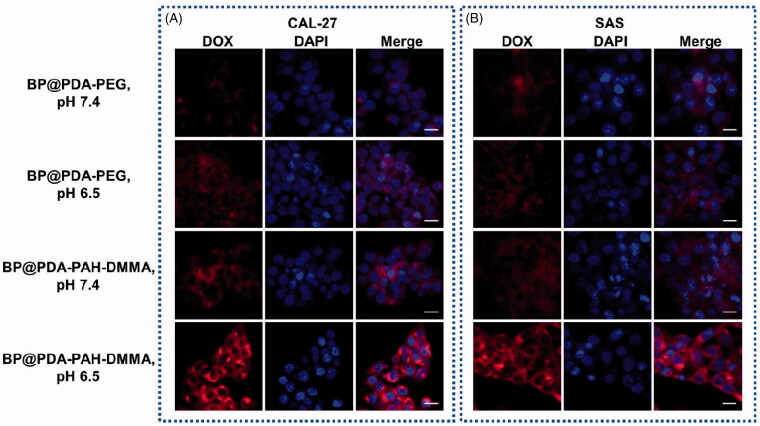
Confocal laser scanning images of oral cancer (A) CAL-27 and (B) SAS cells incubated with DOX labeled BP@PDA-PEG and BP@PDA-PAH-DMMA at different pH values of 7.4 and 6.5 after 4 h. The scale bar is 20 μm.

To evaluate the effect of BP@PDA-PAH-DMMA on tumor cell uptake due to charge reversal in tumor acid environment, doxorubicin (DOX) was used as a fluorescent-labeled dye of different modified BP NSs, and BP@PDA-PEG with a similar size to BP@PDA-PAH-DMMA but no charge reversal effect was synthesized as a control. As can be seen from Figure 6, after 4 h co-incubation with human tongue squamous cell carcinoma cells CAL-27 and SAS, it was observed by confocal laser microscopy that BP@PDA-PAH-DMMA had the strongest red fluorescence intensity in both cells at pH 6.5, and was distributed around the nucleus. However, the only very weak fluorescence signal was observed in the group treated with BP@PDA-PEG or at pH 7.4. This suggested that BP@PDA-PAH-DMMA can greatly promote the uptake of tumor cells under the condition of the weak acid in the tumor microenvironment, which can be attributed to the exposure of a large number of amino groups on PAH due to the break of the dimethylaletic amide bond, so that changed the surface charge from negative to positive.

### 3.6. Cell viability

**Figure 7.**
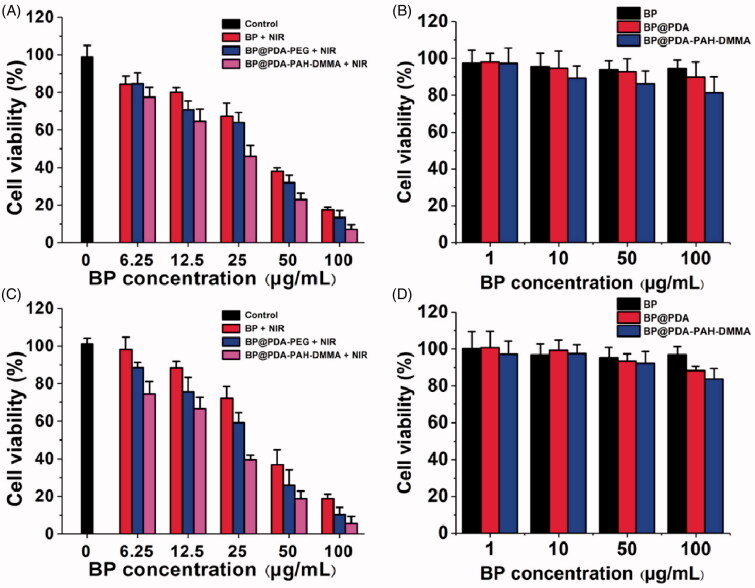
Cytotoxicity of oral cancer (A) CAL-27 and (B) SAS cells cultured with fresh culture medium, BP@PDA-PEG, and BP@PDA-PAH-DMMA under NIR irradiation for 10 min. Viability of (C) CAL-27 and (D) SAS cells cultured with BP NSs, BP@PDA, and BP@PDA-PAH-DMMA without NIR irradiation.

The photothermal cytotoxicity of nanomedicines on CAL-27 and SAS cells was verified by the CCK8 assay. As can be seen from the Figure 7(A, B), irradiation of cells alone does not affect their activity. However, after irradiation under the same conditions with different concentrations of BP, BP@PDA-PEG, and BP@PDA-PAH-DMMA, it can be found that BP@PDA-PAH-DMMA is more lethal to cells than BP@PDA-PEG at the same concentration of BP, which may be caused by the increased uptake of tumor cells due to the PAH-DMMA charge reversal system. The cytotoxicity of BP@PDA-PEG at the same BP concentration was also significantly stronger than that of the bare BP NSs group, which could be attributed to the enhancement of the photothermal properties of the nanomedicine by PDA modification, which matched with the results of *in vitro* photothermal experiments. At the same time, with the increase of BP concentration, the activity of both kinds of cells decreased evidently. Among them, at BP concentration of 50 μg·mL^–1^, less than 40% of CAL-27 or SAS cells survived after different nanomedicine treatments (BP@PDA-PAH-DMMA-treated cells were around 20%). At BP concentration of 100 μg·mL^–1^, the survival rate of both types of cells treated with nanomedicines was less than 20% (BP@PDA-PAH-DMMA-treated cells were less than 10%). Besides, Figure 7(C,D) showed the effects of different concentrations of nanomedicines on cell growth in the absence of 808 nm irradiation. The results showed that more than 80% of the CAL-27 or SAS cells survived even when co-incubated nanomedicines with a BP concentration of 100 μg·mL^–1^. Taken together, BP@PDA-PAH-DMMA has excellent biocompatibility and NIR-response ability for oral cancer cell killing, which meets the treatment needs of clinical oral cancer patients.

### 3.7. *In vivo* infrared thermal images

**Figure 8.**
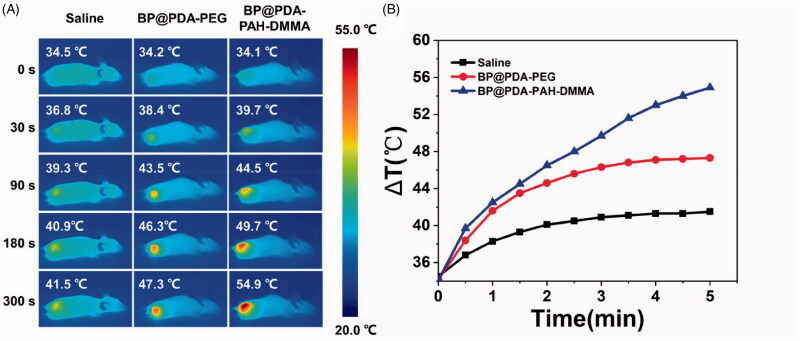
(A) *In vivo* infrared thermal images of CAL-27 tumor-bearing mice after tail vein injection of saline, BP@PDA-PEG and BP@PDA-PAH-DMMA for 24 h, followed by irradiated under 808 nm laser (1.5 W·cm^–2^) for 5 min; (B) Time-dependent temperature rise curves of CAL-27 tumor-bearing mice recorded by an infrared thermal imaging camera under 808 nm laser (1.5 W·cm^–2^) for 5 min.

The photothermal heating effect of nanomedicines *in vivo* was evaluated by infrared imaging after 808 nm NIR irradiation on mice with subcutaneous xenograft of CAL-27 cells. Saline, BP@PDA-PEG, and BP@PDA-PAH-DMMA were injected into two kinds of tumor-bearing mice through a tail vein, respectively. One day later, 808 nm NIR (1.5 W·cm^–2^) was irradiated for 5 min. As can be seen from the infrared thermal imaging images (Figure 8(A)), the temperature change of the BP@PDA-PAH-DMMA group was more dramatic than that of the BP@PDA-PEG group in CAL-27 tumor-bearing mice, which was because the charge reversal of BP@PDA-PAH-DMMA in the tumor site promoted its enrichment in tumor tissues and uptake by tumor cells. Specifically, the final temperature of the tumor site in CAL-27 tumor-bearing mice could reach 54.9 °C, which is high enough to induce tumor cell apoptosis to have a favorable therapeutic effect. Besides, it is worth noting that in the process of NIR irradiation, only the temperature in the local tumor of mice increased sharply while the temperature in other parts remained basically unchanged, which is very conducive to the treatment of local solid tumor of oral cancer and to minimize the damage to normal tissue cells.

## 4. Conclusions

In conclusion, BP@PDA-PAH-DMMA constructed by us had the size suitable for intravenous delivery, the characteristics of enrichment in tumor sites, the ability to promote tumor cell uptake, as well as excellent photothermal properties *in vivo* and *in vitro*, and the killing effect of oral cancer cells, providing a new idea for the treatment of oral cancer. At the same time, due to the simple preparation and treatment processes, the nanomedicine had laid a good foundation for large-scale production and clinical large-scale use, which were expected to make great contributions to the improvement of the cure rate of oral cancer in the future.
